# Sodium Tungstate Promotes Neurite Outgrowth and Confers Neuroprotection in Neuro2a and SH-SY5Y Cells

**DOI:** 10.3390/ijms25179150

**Published:** 2024-08-23

**Authors:** Nora Montero-Martin, María D. Girón, José D. Vílchez, Rafael Salto

**Affiliations:** Department of Biochemistry and Molecular Biology II, School of Pharmacy, University of Granada, E18071 Granada, Spain; nora84@ugr.es (N.M.-M.); damasovr@gmail.com (J.D.V.); rsalto@ugr.es (R.S.)

**Keywords:** sodium tungstate, neurite outgrowth, neuroprotection, myocyte enhancer factor 2 (MEF2), mitogen-activated protein kinase/extracellular signal-regulated kinase (MAPK/ERK), phosphatidylinositol 3-kinase (PI3K/Akt), mechanistic target of rapamycin (mTOR), advanced glycation end products (AGEs)

## Abstract

Sodium tungstate (Na_2_WO_4_) normalizes glucose metabolism in the liver and muscle, activating the Mitogen-activated protein kinase/extracellular signal-regulated kinase (MAPK/ERK) pathway. Because this pathway controls neuronal survival and differentiation, we investigated the effects of Na_2_WO_4_ in mouse Neuro2a and human SH-SY5Y neuroblastoma monolayer cell cultures. Na_2_WO_4_ promotes differentiation to cholinergic neurites via an increased G1/G0 cell cycle in response to the synergic activation of the Phosphatidylinositol 3-kinase (PI3K/Akt) and ERK1/2 signaling pathways. In Neuro2a cells, Na_2_WO_4_ increases protein synthesis by activating the mechanistic target of rapamycin (mTOR) and S6K kinases and GLUT3-mediated glucose uptake, providing the energy and protein synthesis needed for neurite outgrowth. Furthermore, Na_2_WO_4_ increased the expression of myocyte enhancer factor 2D (MEF2D), a member of a family of transcription factors involved in neuronal survival and plasticity, through a post-translational mechanism that increases its half-life. Site-directed mutations of residues involved in the sumoylation of the protein abrogated the positive effects of Na_2_WO_4_ on the MEF2D-dependent transcriptional activity. In addition, the neuroprotective effects of Na_2_WO_4_ were evaluated in the presence of advanced glycation end products (AGEs). AGEs diminished neurite differentiation owing to a reduction in the G1/G0 cell cycle, concomitant with lower expression of MEF2D and the GLUT3 transporter. These negative effects were corrected in both cell lines after incubation with Na_2_WO_4._ These findings support the role of Na_2_WO_4_ in neuronal plasticity, albeit further experiments using 3D cultures, and animal models will be needed to validate the therapeutic potential of the compound.

## 1. Introduction

Neuronal plasticity is an essential process during development, where neurite outgrowth is needed to constitute efficient networks of neurons [1]. Furthermore, the capability to outgrow neurites is a key step in the neuronal regeneration process [2]. After an injury, the stimulation of several signaling pathways leads to neurite outgrowth. Furthermore, drug-targeted neurite outgrowth is proposed as a promising strategy against neurodegenerative diseases such as Alzheimer’s and Parkinson’s. Also, the neurite outgrowth constitutes the basis of neuronal plasticity needed to maintain daily brain functionality [1,2]. Neurite outgrowth is mainly regulated by two signaling pathways, PI3K/Akt and the mitogen-activated protein kinase/extracellular signal-regulated kinase (MAPK/ERK) pathways [3,4]. Due to the relevance of the process, nowadays there is an active search for neurotrophic molecules, able to penetrate the blood–brain barrier, and with favorable bioavailability [5]. For this search, several in vitro models for the study of neurite differentiation have been used, including Neuro2a [6,7,8] and SH-SY5Y [9,10,11] cell lines.

Sodium tungstate (Na_2_WO_4_) is an oral hypoglycemic agent with low or no toxicity administered orally [12]. The tissues where Na_2_WO_4_ exerts its glucose-normalizing properties are the liver [13], muscle [14], and pancreas [15]. In these tissues, the glucose-normalizing activity relies on the activation of the MAPK/ERK pathway, independently of the presence of insulin [16]. Furthermore, Na_2_WO_4_ can cross the blood–brain barrier [17], making this small molecule an attractive compound that modulates physiological and pathological processes in other tissues and cell types. 

Therefore, this work aims to evaluate the effects of Na_2_WO_4_ on neurite outgrowth and neuroprotection in two neuroblastoma cells and to unravel the underlying molecular mechanisms of its effects. Our findings support the involvement of this compound in neurotrophic effects through the activation of Akt and ERK1/2 signaling pathways and an increase in MEF2D levels.

## 2. Results and Discussion

The use of in vitro model systems has greatly improved neurobiology and neuroscience. Cells in culture allow the characterization of the functions of proteins and genes and the molecular mechanisms underlying specific phenomena, thus permitting us to understand the pathology of diseases and perform preliminary evaluations of pharmacological trials. In neurobiology, the most commonly used cell culture models include rat- and mouse-derived primary neuronal cultures and neuroblastoma cell lines, such as rat B35, mouse Neuro2a [6,7,8], rat PC12, and human SH-SY5Y cells [9,10,11]. 

Neuro2a and SH-SY5Y cells undergo partial differentiation when grown in a differentiation medium with a restriction of FBS. We selected these two cell lines as models to study the effects of sodium tungstate (Na_2_WO_4_) on neurite proliferation and differentiation and the molecular bases involved in these processes. By using two cell lines, we avoided the effects that could be due to the specific idiosyncrasy of one cell line. All results were similar in Neuro2a and SH-SY5Y cells.

**Figure 1:**
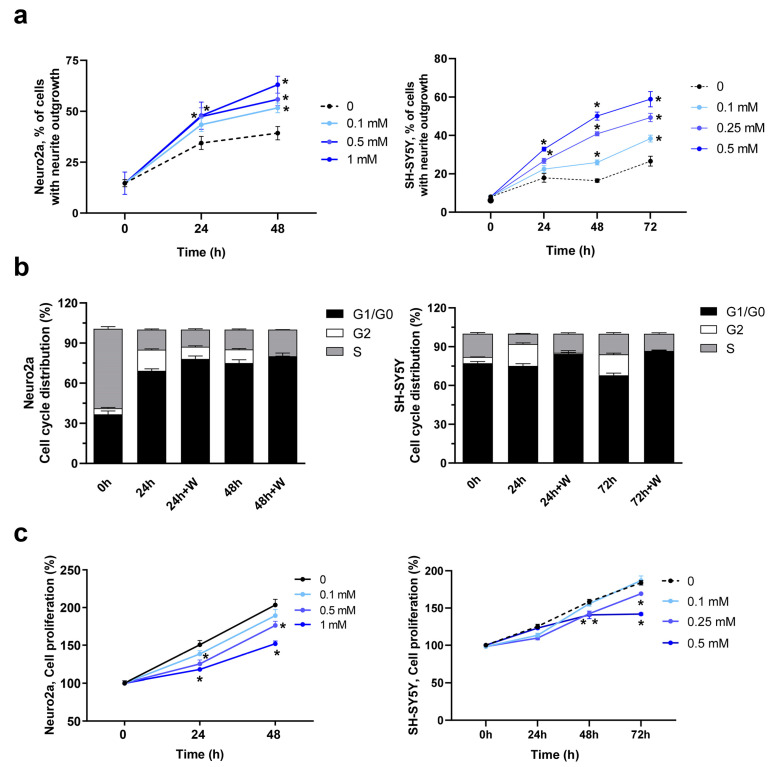
Na_2_WO_4_ induces neurite outgrowth in Neuro2a (**left column**) and SH-SY5Y (**right column**) cells. Neuro2A and SH-SY5Y cells were incubated with increasing concentrations of Na_2_WO_4_, and neurite outgrowth (plotted as the percentage of neurite-bearing cells to total cells) (**a**), cell cycle, for the G1/G0 and G2 values there are significant differences between control cells and Na_2_WO_4_ treated cells at each incubation time (**b**), and cell viability (plotted as the percentage of viability at time 0) (**c**) were measured at different time points. Data are mean ± SEM (*n* = 8). * *p* < 0.05 of Na_2_WO_4_ incubated cells vs control cells.

Na_2_WO_4_ is a well-known activator of the ERK1/2 signaling pathway in skeletal muscle [14,18] and liver [13], and since this pathway is also involved in neurite outgrowth [19], the effects of Na_2_WO_4_ on cell differentiation, cell cycle, and cell proliferation were analyzed in Neuro2a and SH-SY5Y cells incubated in differentiation media (Figure 1). We visualized the morphological changes after 48 or 72 h of Na_2_WO_4_ supplementation. It can be observed that while most untreated cells showed a rounded shape without neurite extension, extensive neurite formation was observed in Na_2_WO_4_-treated cells using phase-contrast microscopy (Figure 1a and Supplementary Materials Figure S1). 

The Na_2_WO_4_-induced neurite outgrowth was concentration-dependent in both cell types (Figure 1a). The Na_2_WO_4_ dose with a significant response was 1 mM in Neuro2a cells and 0.25 mM in SH-SY5Y cells. Likewise, the incubation times required to obtain these effects in neurite outgrowth are 24–48 or 72 h for Neuro2a and SHSY5Y cells, respectively. These doses of Na_2_WO_4_ and incubation times were used in the remaining experiments.

The formation of the central nervous system requires an initial period of high progenitor cell proliferation followed by differentiation. Thus, differential growth and cellular diversity occur in different regions of the central nervous system. Neurite differentiation involves the growth and maturation of dendrites and the axons of developing neurons. The process is initiated by the emission of one or more primary buds from the cell soma. The cell cycle exits the quiescent G0 phase, which is the fundamental step in triggering cell differentiation and inducing a new gene expression program leading to the elaboration of a specialized phenotype [20,21].

To determine whether the differentiation observed in Na_2_WO_4_-treated cells is related to changes in cell cycle progression, the two neuroblastoma cell lines were incubated in the absence or presence of an effector. Incubation with Na_2_WO_4_ for 48 h (Neuro2a cells) or 72 h (SH-SY5Y cells) promotes an increase in the G1/G0 phase parallel to a decrease in the G2 phase, indicating entry into the differentiation process. Neural progenitor cells have a longer cell cycle before entering differentiation and are generally attributed to an extended G1 phase as progenitors switch from a mode of proliferative division to neuritogenesis [22]. Furthermore, in the presence of Na_2_WO_4,_ the synthesis phase was maintained to allow active neurite outgrowth (Figure 1b and Supplementary Materials Figure S2).

**Figure 2:**
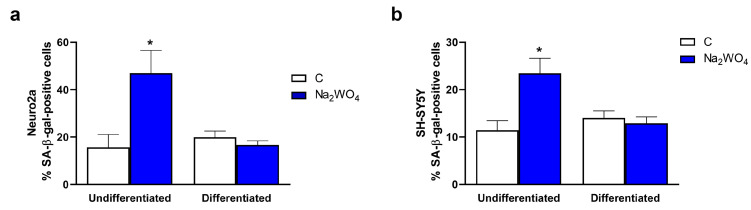
Na_2_WO_4_ increased the number of undifferentiated SA-β-gal-positive cells. Cells were differentiated for 48 h in the presence or absence of 1 mM (Neuro2a) or 0.25 mM (SH-SY5Y) Na_2_WO_4_ and the percentage of SA-β-gal-positive cells was measured as described in Section 3. (**a**) The percentage of Neuro2a SA-β-gal-positive cells. (**b**) The percentage of SH-SY5Y SA-β-gal-positive cells. Results represent means ± SEM (*n* = 4). * *p* < 0.05 vs untreated cells.

Figure 2 shows the percentage of Neuro2a- or SH-SY5Y SA-β-gal-positive cells. Na_2_WO_4_ increased the senescence of undifferentiated cells, whereas it did not affect the senescence levels of differentiated cells compared with control cells.

Linked with the Na_2_WO_4_-mediated increase in neurite outgrowth in these cells, slower cell proliferation was detected (Figure 1c) and was not related to any significant effect on cell death measured by lactic dehydrogenase activity release (Supplementary Material Figure S3). It should be considered that differentiated cells cannot grow, which partially explains the Na_2_WO_4_-mediated decrease in proliferation. Furthermore, we explored other possible mechanisms involved in slower cell proliferation. Cellular senescence [23] is characterized by permanent cell cycle arrest and apoptosis resistance. Enhanced senescence in cells differentiated from nervous tissue is associated with neurodegenerative diseases. Therefore, we investigated whether changes induced by Na_2_WO_4_ in neurite outgrowth were concomitant with permanent cell cycle arrest and senescence in the non-differentiated population of cells. To test this hypothesis, Neuro2a cells were incubated with or without Na_2_WO_4_, and senescence was analyzed using the β-galactosidase assay as a senescence marker (Figure 2). 

**Figure 3:**
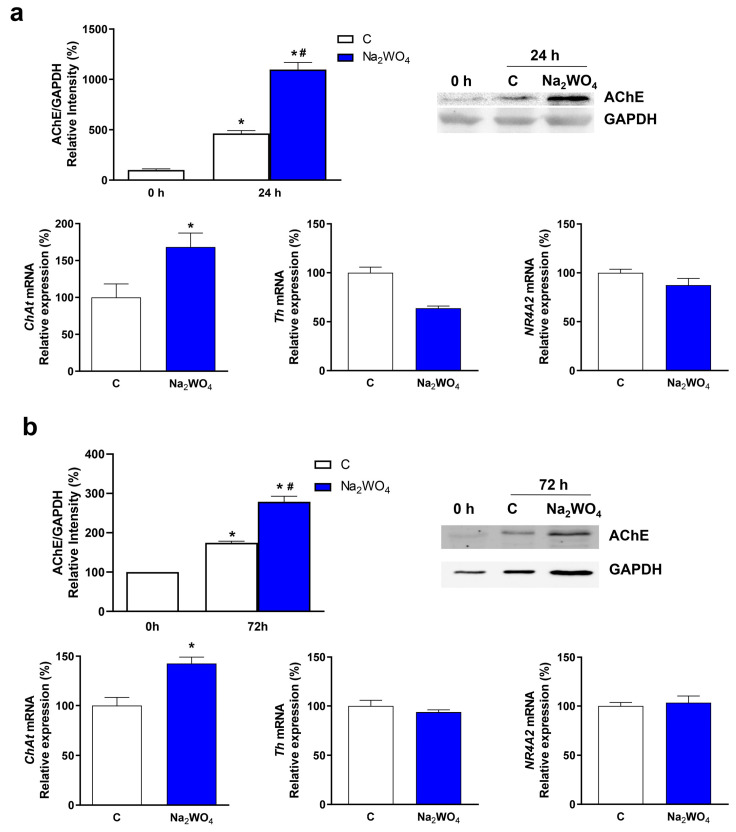
Na_2_WO_4_ induces the expression of the molecular markers of neuronal differentiation toward a cholinergic phenotype. (**a**) Neuro2a cells were incubated in a differentiation medium in the absence or presence of 1 mM sodium tungstate for 24 h. (**b**) SH-SY5Y cells were incubated in a differentiation medium in the absence or presence of 0.25 mM sodium tungstate for 72 h. Acetylcholinesterase expression (AChE) was measured by Western blotting using a specific antibody against AChE. Results were normalized using GAPDH as the loading control. As described in Section 3, the mRNA levels of the *choline O-acetyltransferase* (*ChAt*), *tyrosine hydroxylase* (*Th*), and *NR4A2* genes were measured by qPCR. Results are expressed as mean ± SEM (*n* = 6). * *p* < 0.05 vs control cells at 0 h. # *p* < 0.05 between control and Na_2_WO_4_ treated cells at the final incubation time.

Taken together, our results indicate that Na_2_WO_4_ increases neurite outgrowth without producing any unwanted proliferation, and its activity could be of interest to induce neuronal plasticity. To further investigate differentiation mediated by Na_2_WO_4_, markers of neurite differentiation were analyzed (Figure 3). 

The effects of Na_2_WO_4_ on neurite formation translate into the overexpression of molecular markers of neuronal differentiation toward the cholinergic phenotype in both cell types (Figure 3). Acetylcholinesterase (AChE) expression was significantly increased in the Na_2_WO_4_-incubated cells in Neuro2a and SH-SY5Y cells. Moreover, when the expression of the gene *choline O-acetyltransferase* (*ChAt*), which encodes an enzyme that catalyzes the biosynthesis of the neurotransmitter acetylcholine, was measured, an increase in the levels of ChAt was observed in the Na_2_WO_4_-incubated cells. 

To further confirm Na_2_WO_4_-induced neurite outgrowth was directed to cholinergic cells in Neuro2a cells, the expression of the *tyrosine hydroxylase* (*Th*) and *NR4A2* genes, which are essential for the development of dopaminergic neurons, was measured by RT-qPCR. Incubation with Na_2_WO_4_ did not modify the expression of these two genes in both cell lines.

Increased neurite differentiation in the Neuro2a cell line has been classically used as an in vitro model of long-term memory formation [24]. Therefore, this result warrants further study to elucidate the molecular mechanism of action of Na_2_WO_4_ in the neurite outgrowth process.

**Figure 4:**
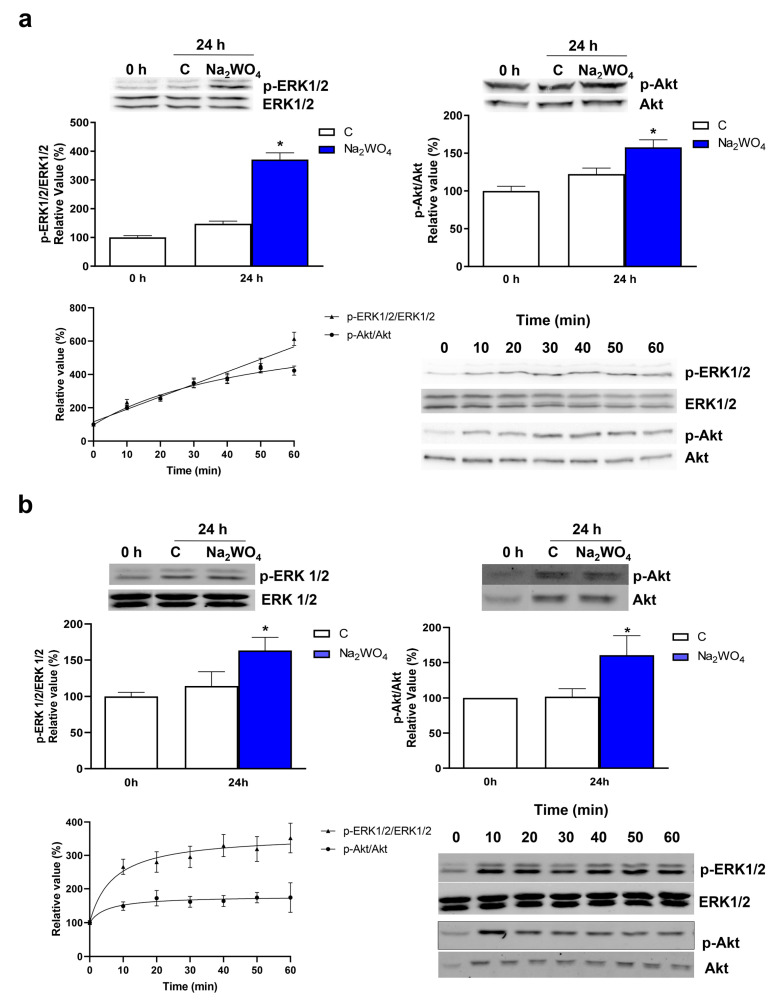
Na_2_WO_4_ activates the PI3K/Akt and ERK1/2 signaling pathways in Neuro2a and SH-SY5Y cells. Na_2_WO_4_ was added to Neuro2a (**a**) or SH-SY5Y (**b**) cells for 24 h or from 0 to 60 min to determine the phosphorylation time course. Western blotting was performed using specific antibodies against phosphorylated and total Akt and ERK1/2. Results are expressed as mean ± SEM (*n* = 6). * *p* < 0.05 vs untreated cells.

It has been shown that the activation of the PI3K/Akt signaling pathway plays a key role in neuronal outgrowth [3,4,19]. In Neuro2a cells, ERK1/2 signaling activation is also relevant to neuronal differentiation. For example, α-lipoic acid [25], lithium [26], and HMB [19] participate in neurite outgrowth activating the ERK1/2 signaling pathway. In addition, previous studies demonstrated that Na_2_WO_4_ activates ERK1/2 but not Akt signaling in the liver [13] or skeletal muscle [14,18]. The activation of the ERK1/2 pathway by Na_2_WO_4_ stimulates glucose transport and glycogen synthesis in muscle and liver cells and improves protein turnover. We analyzed the effects of Na_2_WO_4_ on Akt and ERK1/2 signaling in Neuro2a and SH-SY5Y cells by measuring the expression of the phosphorylated forms of PKB/Akt and ERK1/2 in Neuro2a (Figure 4a) and SH-SY5Y (Figure 4b) by immunoblotting after treating the cells with or without Na_2_WO_4_ for 24 h. As shown in Figure 4, Na_2_WO_4_ increased ERK1/2 and PKB/Akt phosphorylation in both cell lines. The activation of the Akt pathway by Na_2_WO_4_ is a novel result that we have only identified in nervous system-derived cell lines. To confirm the activation of both signaling pathways, we studied the effects of Na_2_WO_4_ over short periods. Our results indicate that the Na_2_WO_4_ activation of both pathways and cell lines is produced in 15–30 minutes, and the phosphorylation remains after 1 h.

**Figure 5:**
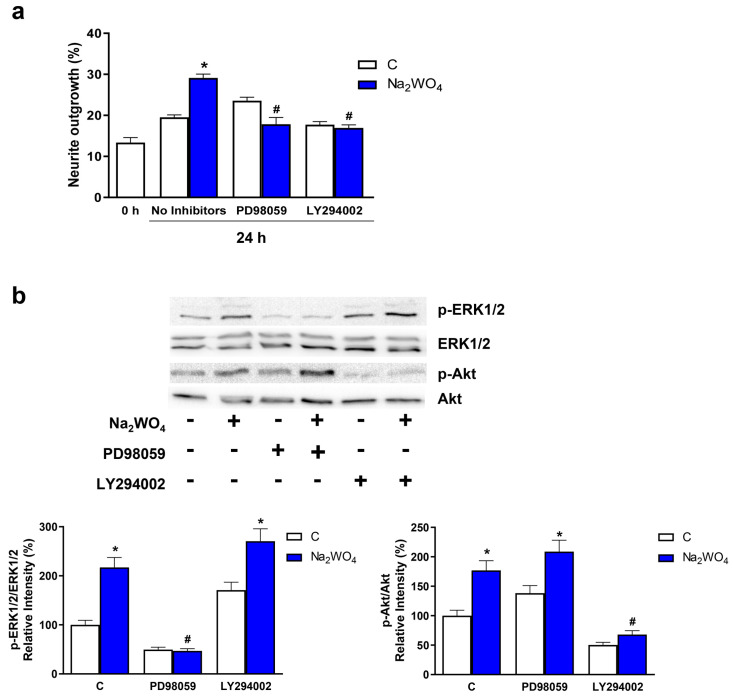
Na_2_WO_4_-induced neurite outgrowth is mediated by the PI3K/Akt and ERK1/2 signaling pathways in Neuro2a cells. Neuro2a cells were pre-treated with PD98059 10 μM or LY294002 20 μM and then treated with 1 mM Na_2_WO_4_ for 24 h (**a**) or 30 min (**b**). Inhibitors were maintained during the experiment. (**a**) The percentage of differentiated cells was determined by analyzing cell morphology (Supplementary Materials Figure S4). (**b**) ERK1/2 and Akt activation were measured by Western blotting, as shown in Figure 4. Results represent means ± SEM (*n* = 4). * *p* < 0.05 vs untreated cells; # *p* < 0.05 vs Na_2_WO_4_ treated cells without inhibitors.

To determine whether the activation of these kinases by Na_2_WO_4_ was involved in the regulation of neuritogenesis, Neuro2a cells were pre-incubated with inhibitors of Akt (20 μM LY294002) and ERK1/2 (10 μM PD98059). The selective blockade of the activation of both kinases was confirmed by Western blotting. Our results (Figure 5a) indicate that in the absence of Na_2_WO_4_, neurite outgrowth requires the activation of the Akt signaling pathway independently of ERK1/2 signaling. On the contrary, the positive effects of Na_2_WO_4_ on neurite outgrowth require that both signaling pathways remain active, providing strong evidence of the synergistic involvement of both signaling pathways in the Na_2_WO_4_-induced neurite outgrowth.

**Figure 6:**
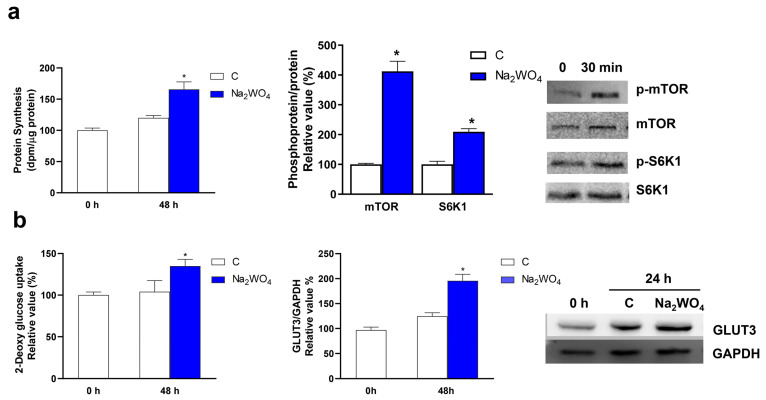
Na_2_WO_4_-induced neurite outgrowth is concomitant with increased protein synthesis and glucose uptake in Neuro2a cells. Neuro2a cells were incubated with 1 mM Na_2_WO_4_ for 24 h. Then, protein synthesis (**a**) and 2-deoxyglucose uptake (**b**) were measured as described in Section 3. Additionally, GLUT3 levels were determined using Western blotting. The phosphorylation status of mTOR and S6K1 was measured after 30 min incubation with 1 mM Na_2_WO_4_. Results represent means ± SEM (*n* = 4). * *p* < 0.05 vs untreated cells.

For neurite formation, it is necessary for a series of metabolic adaptations to provide the cell with metabolic fuels and increased protein synthesis [27]. Therefore, protein synthesis was measured in Neuro2a cells together with the activation of mTOR and S6K, two kinases involved in the beginning of translation and whose activation is also dependent on the phosphorylation of Akt and/or ERK1/2 [19]. The incubation of Neuro2a cells with Na_2_WO_4_ induced a significant increase in protein synthesis and the activation of mTOR and S6K (Figure 6a).

During neuronal differentiation, an increase in glucose metabolism is required based on enhanced glucose uptake mediated by the GLUT3 transporter. In neurons, aerobic glycolysis is needed for maintaining axonal elongation and synaptogenesis. This pathway provides energy for protein synthesis and intermediaries, such as acetyl-CoA, that contribute to lipid synthesis and membrane expansion during neurite growth. This metabolic reprogramming is mediated by the activation of the PI3K/mTOR axis [28,29,30]. Our results (Figure 6b) indicate that Na_2_WO_4_ increases the expression of the GLUT3 transporter in Neuro2a cells to maintain neuritogenesis and cell survival. 

**Figure 7:**
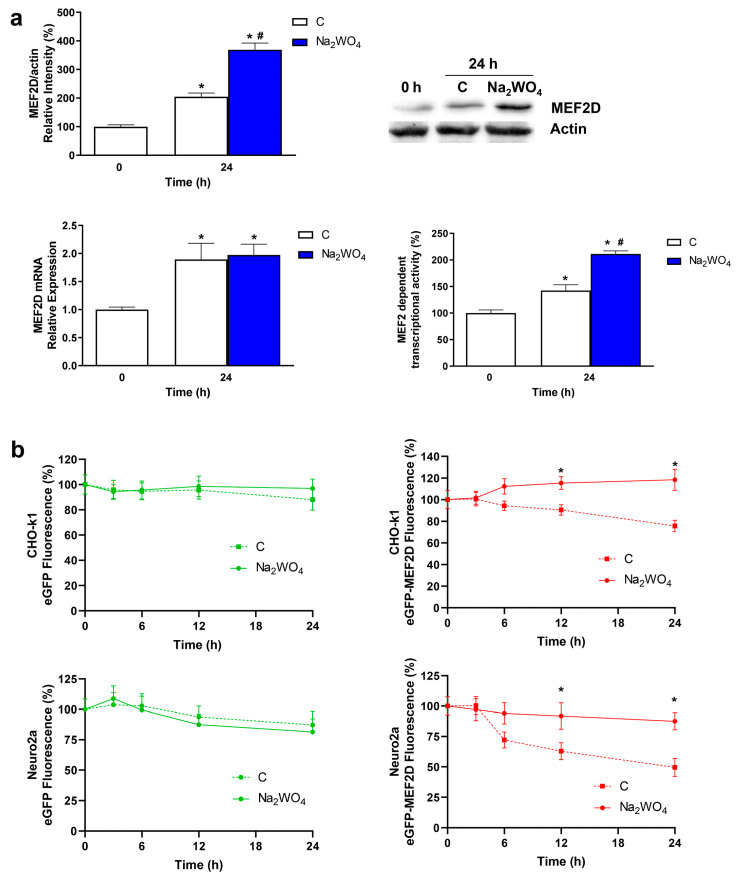
Na_2_WO_4_ increased the expression of the transcription factor MEF2D in Neuro2a cells. (**a**) Neuro2a cells were incubated with 1 mM Na_2_WO_4_ for 24 h, and the protein and mRNA expression levels of MEF2D were measured. The transcriptional activity dependent on the MEF2 promoter was evaluated using pMEF2x4 Eb1 Luc, a luciferase reporter system that contains MEF2 binding sites. (**b**) CHO-k1 or Neuro2a cells were transfected with plasmids that encode for eGFP or a fusion protein between eGFP and MEF2D. Transfected cells treated or not with 1 mM Na_2_WO_4_ were then incubated with 100 µM cycloheximide for 24 h, and eGFP fluorescence was measured as described in Section 3. Results represent means ± SEM (*n* = 4). * *p* < 0.05 vs 0 h control cells; # *p* < 0.05 vs 24 h control cells.

The MEF2 transcription factor family participates in the survival and differentiation of several types of neurons. In mice, brain-specific triple deletion of MEF2A/C/D resulted in early postnatal lethality and increased neuronal apoptosis, resulting in an intensified neuroinflammatory response and brain injury. In contrast, MEF2D overexpression inhibited microglial activation, reduced cytokine levels, and protected neurons against oxygen and glucose deprivation. These observations suggest that MEF2D plays a critical role in modulating inflammatory responses and providing neuroprotection in the context of brain injury [31,32,33]. Furthermore, MEF2 expression depends on Akt and ERK1/2 signal transduction [34]. In muscle cells, Na_2_WO_4_ increases the levels of MEF2D to promote glucose uptake through the increase in the GLUT4 transporter at the plasma membrane [14]. Therefore, we analyzed whether the MEF2 family is involved in the effects of Na_2_WO_4_ on neurite outgrowth (Figure 7). 

In Neuro2a cells, Na_2_WO_4_ increased the expression of the MEF2D protein (Figure 7a). Moreover, Na_2_WO_4_-incubated cells showed higher transcriptional activity than untreated cells using a reporter gene construction that contains 4 repeats of the binding sequence of MEF2 fused to pGL3 (pMEF2x4 Eb1 Luc). This result supports the positive effects of Na_2_WO_4_ on neurite outgrowth, as an increase in MEF2D levels was detected during neuronal differentiation, and a positive correlation has been described between MEF2D levels and neurite length in rats [35]. 

Interestingly, although Na_2_WO_4_ increases MEF2D protein levels in Neuro2a cells, no changes in its mRNA levels were detected (Figure 7a), indicating post-transcriptional regulatory mechanisms supporting the effects of Na_2_WO_4_, either by an increase in protein translation or a decrease in protein degradation. To elucidate these effects, we designed experiments to analyze the Na_2_WO_4_ effects on MEF2D protein half-life. To achieve this, we used a eukaryotic expression plasmid that encodes the rat MEF2D protein. This protein is highly homologous to mouse and human proteins and maintains all regulatory sequences [36]. Cells that did not express (CHO-k1 cells) or express MEF2D (Neuro2a cells) were transfected with a plasmid that encodes for the eukaryotic green fluorescent protein (eGFP) or eGFP fused to the rat MEF2D wild-type protein under the control of a CMV promoter. Then, we performed a cycloheximide (an inhibitor of protein synthesis) chase assay to measure steady-state protein stability (Figure 7b). Because eGFP is a relatively stable protein, in untreated or Na_2_WO_4_-treated cells, the fluorescence associated with cells did not decrease after 24 h. On the contrary, the fluorescence of cells transfected with the plasmid encoding the eGFP-MEF2D fusion protein significantly declined during the 24 h in untreated cells, whereas in cells treated with Na_2_WO_4_, the fluorescence values remained unchanged during all treatments. These results support the theory that Na_2_WO_4_ increases MEF2D expression by decreasing protein degradation.

Expression and transcriptional activity of the MEF2 family are regulated by phosphorylation, sumoylation, and acetylation [37]. Human and rat MEF2D are sumoylated at Lys439, initiating a protein degradation process. A phosphorylable serine residue at position 444, which is highly conserved among MEF2 proteins, appears to regulate sumoylation and thus protein degradation. When Cdk5, a cyclin-dependent kinase, phosphorylates S444, the transcriptional activity of MEF2D decreases and sumoylation is stimulated [37].

**Figure 8:**
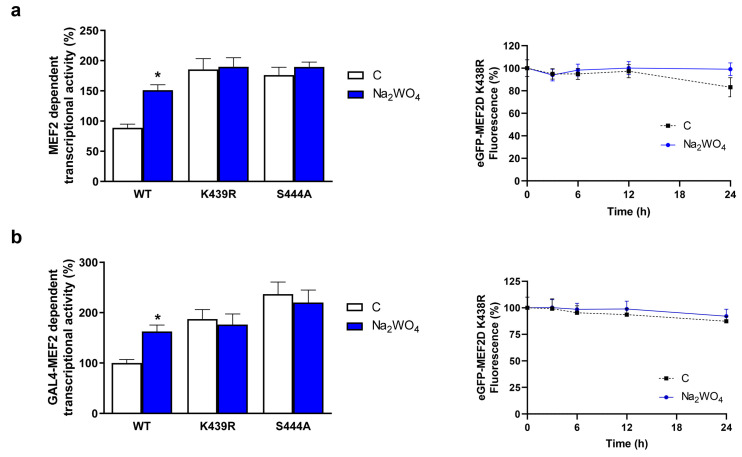
Na_2_WO_4_ modulates MEF2D protein stability. (**a**) CHO-k1 cells were transfected with a plasmid that either expressed mutated or wild-type MEF2D proteins and a plasmid that encoded a luciferase reporter system that contained MEF2 binding sites. (**b**) Neuro2a cells were co-transfected with a plasmid that either expressed mutated or wild-type MEF2D fused to the GAL4 DNA binding domain and a plasmid encoding a luciferase reporter system under the control of 4 GAL4 binding sites. CHO-k1 and Neuro2a cells were incubated with 1 mM Na_2_WO_4_ for 24 h and transcriptional activity was evaluated. In addition, CHO-k1 (**a**) and Neuro2a (**b**) cells were transfected with plasmids encoding eGFP or a fusion protein between eGFP and wild-type or mutant MEF2D. Transfected cells were treated or not treated with 1 mM Na_2_WO_4_ and then incubated with cycloheximide for 24 h, and eGFP fluorescence was measured as described in Section 3. Results represent means ± SEM (*n* = 4). * *p* < 0.05 vs untreated cells.

We indirectly studied the effects of Na_2_WO_4_ on the regulation of the transcriptional activity and sumoylation of the MEF2D protein (Figure 8). First, we studied the MEF2D-dependent transcriptional activity in cells that do not express MEF2D (CHO-k1 cells) using the reporter plasmid pMEF2x4 Eb1 Luc (that contains MEF2 binding sites). These cells were also co-transfected with plasmids expressing different MEF2D mutants. We used constructions in which K439 was mutated to Arg (MEF2D K439R) and plasmids expressing a mutation at position 444 (MEF2D S444A). The mutation at position 439 prevents sumoylation, and the S444A mutation blocks the phosphorylation at position 444 that regulates sumoylation. Our results (Figure 8a) show that while Na_2_WO_4_ increased wild-type MEF2-dependent transcription when CHO-k1 cells were co-transfected with constructs expressing MEF2 K439R or MEF2 S444A, no differences were found in the transcriptional activity of MEF2 between untreated and treated cells. In addition, when in a cycloheximide chase experiment, the eGFP fluorescence of cells transfected with the eGFP-MEF2 K439R mutant was analyzed, no changes in fluorescence upon incubation with Na_2_WO_4_ were detected up to 24 h compared with the untreated cells.

Although the experiments indicated that the Na_2_WO_4_ effects on MEF2D protein levels were due to the regulation of protein sumoylation, additional experiments (Figure 8b) were carried out in Neuro2a cells to confirm that these effects remained in place in a neuroblastoma cell line background. Because these cells express endogenous MEF2D, a strategy was designed to avoid the influence of the wild-type endogenous protein on the analysis of the mutated versions of MEF2D. For this purpose, we used a reporter plasmid (pFR-Luc) containing copies of the GAL4 binding sequence [38]. Neuro2a cells were co-transfected with pFR-Luc and plasmids encoding fusion proteins between the GAL4 DNA-binding domain and wild-type MEF2D or its mutated versions (GAL-MEF2D K439R, and GAL-MEF2D S444A). The analysis of transcriptional activity in Neuro2a cells produced similar results to those of CHO-k1 cells. Furthermore, measurement of the eGFP-MEF2 K439R half-life in Neuro2a cells provides the same results as in non-expressing MEF2D CHO-k1 cells, confirming that the residue at position 439 plays a central role in the regulation of the effects of Na_2_WO_4_. The identification of kinases that are downregulated by Na_2_WO_4_ and responsible for the S444 phosphorylation remains to be elucidated. Albeit, in muscle, we have described that Na_2_WO_4_ increased MEF2D levels [14]. Here, we expand these findings to neurites and elucidate the molecular basis of this effect.

Next, after determining the effects on Na_2_WO_4-_mediated neurite outgrowth, we tested if Na_2_WO_4_ can confer neuroprotection in an in vitro model. For this purpose, we selected a model in which advanced glycation end products (AGEs) were used as the stressor agent. AGEs are by-products of diabetes and are produced when proteins react with sustained high blood glucose levels. In neurites, AGEs, through their interaction with their receptor, produce a re-entry in the cell cycle, blocking neurite differentiation and increasing cell proliferation [39,40]. In addition, in Neuro2a, AGEs can imbalance the redox state and induce the activation of caspase−3 activity and apoptosis [41].

**Figure 9:**
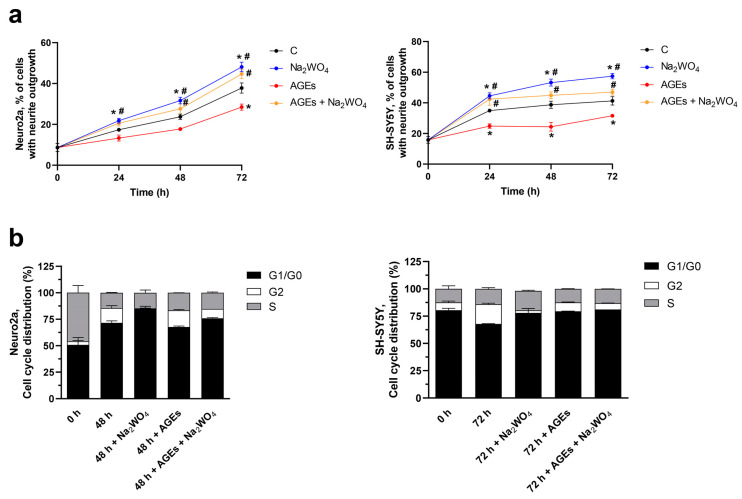
Na_2_WO_4_ reversed the effects of advanced glycation end products (AGEs) on neurites. Neuro2a and SH-SY5Y cells were incubated with 100 µg/mL of AGEs in the absence or presence of Na_2_WO_4_. (**a**) Percentage neurite outgrowth in Neuro2a or SH-SY5Y cells incubated with the effectors. Results represent means ± SEM (*n* = 4). * *p* < 0.05 vs untreated cells; # *p* < 0.05 vs AGEs treated cells. (**b**) Analysis of the cell cycle, for the G1/G0 and G2 values there are significant differences between control cells and Na_2_WO_4_-treated cells.

In Neuro2a and SH-SY5Y cells, Na_2_WO_4_ (Figure 9a and Supplementary Materials Figure S5) increased neurite outgrowth compared with the untreated control cells, and as expected, incubation with AGEs in the absence of Na_2_WO_4_ significantly reduced neuritogenesis. On the contrary, incubation of cells with Na_2_WO_4_ in the presence of AGEs restored neurite formation to levels like those observed in cells treated with Na_2_WO_4_. Figure 9b presents the cell cycle in AGEs-treated cells. As shown in Figure 1b, Na_2_WO_4_ promoted an increase in the G1/G0 phase compared with the G2 phase, indicating its entrance into the differentiation process. Incubation with AGEs for 48 or 72 h decreased the percentage of cells in the G1/G0 phase and increased G2, suggesting re-entry into the cell cycle that further prevented neurite differentiation [39,40]. On the contrary, incubation with Na_2_WO_4_ in the presence of AGEs increased the G1/G0 phase, resulting in a situation like that of Na_2_WO_4_-treated cells.

**Figure 10:**
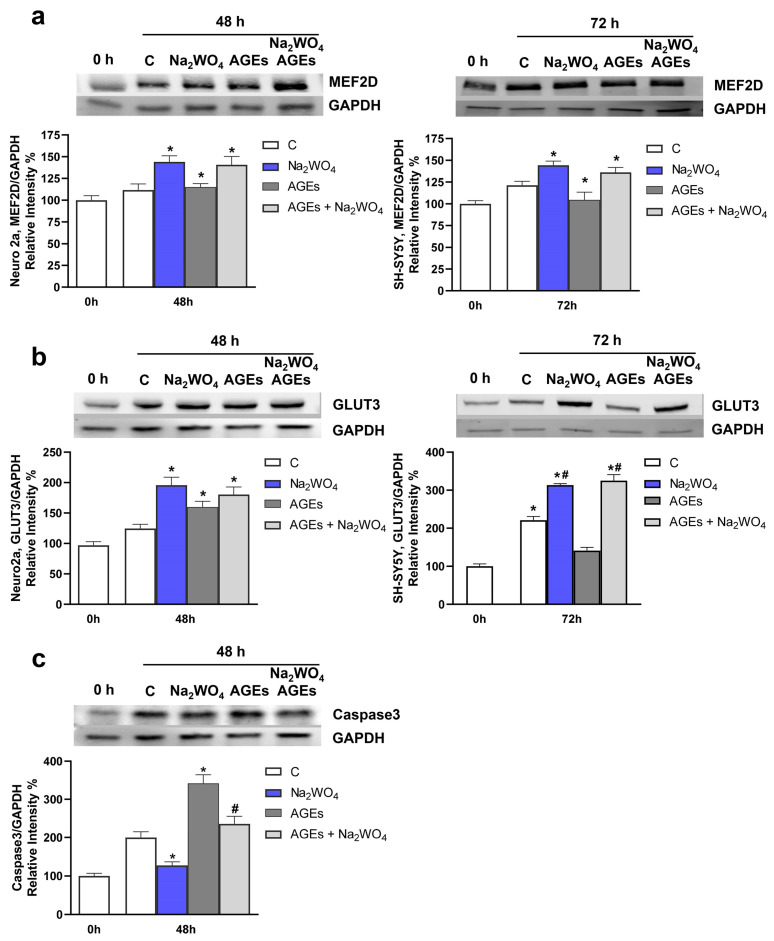
Na_2_WO_4_ protects against advanced glycation end products (AGEs) effects on neurites. Neuro2a and SH-SY5Y cells were incubated with 100 µg/mL of AGEs in the absence or presence of Na_2_WO_4_. (**a**) MEF2D expression in Neuro2a and SH-SY5Y cells. (**b**) GLUT3 transporter expression in Neuro2a and SH-SY5Y cells. (**c**) Processed caspase−3 levels in Neuro2a cells incubated with the effectors. Results represent means ± SEM (*n* = 4). * *p* < 0.05 vs untreated cells; # *p* < 0.05 vs AGE-treated cells.

Next, because Na_2_WO_4_ increases MEF2D expression in neurites, the protein levels of the transcription factor were assayed in the presence of AGEs and Na_2_WO_4_. Age and AGE deposits are associated with decreased neuronal growth factors [42]. Confirming these results, our experiments indicate that incubation with AGEs in Neuro2a and SH-SY5Y cells translates into a decrease in the expression of MEF2D. In contrast, incubation with Na_2_WO_4_ prevented the negative effects of AGEs (Figure 10a). Furthermore, considering the relevance of GLUT3-mediated glucose uptake in neurons and its influence on ameliorating aging [29], we measured the levels of this transporter in the presence of AGEs and in combination with Na_2_WO_4_ (Figure 10b). Incubation with AGEs decreased the expression of GLUT3, as previously described [43]. Again, incubation in the presence of Na_2_WO_4_ blocked the negative effects of AGEs in both cell lines.

Finally, incubation with AGEs increased apoptosis in neurons [42]. This effect is partially mediated by the proteolytic activation of caspase−3 [41]. In Neuro2a cells, we measured the activation of caspase−3 using Western blotting, and as expected, incubation with AGEs alone increases the amount of processed caspase. In contrast, this processing was prevented by incubation in the presence of Na_2_WO_4_ (Figure 10c and Supplemental Materials Figure S7). 

In conclusion, Na_2_WO_4_ is a well-known glucose-normalizing therapeutic agent that can cross the brain barrier and exert neurotropic and neuroprotective effects by activating specific signaling pathways. These effects are mediated synergically by Akt and ERK1/2 signaling pathways that translate into an enhanced neurite outgrowth, differentiation to cholinergic neurites, and stimulation of cell metabolism. Na_2_WO_4_ decreases the degradation of the neuronal-specific transcription factor MEF2D. Further experiments using neural organoids or other 3D culture models, and animal experiments are needed to validate the therapeutic potential of the compound.

## 3. Materials and Methods

### 3.1. Cell Culture

The murine neuroblastoma Neuro2a (N2a; ATCC No. CCL−131) cell line was grown in Dulbecco’s Modified Eagle’s medium (DMEM) supplemented with 10% (*v*/*v*) FBS, 2 mM glutamine plus 100 U/mL penicillin, and 0.1 mg/mL streptomycin in an atmosphere of 5% CO_2_ and 95% air. It was maintained at subconfluent densities in the growth medium. For cell proliferation experiments, we used DMEM supplemented with 10% FBS. The medium was replaced with DMEM supplemented with 0.5% FBS for differentiation experiments.

The human neuroblastoma SH-SY5Y (ECACC No. 94030304) cell line was grown in DMEM supplemented with 15% (*v*/*v*) FBS, 2 mM glutamine plus 100 U/mL penicillin, and 0.1 mg/mL streptomycin in an atmosphere of 5% CO_2_ and 95% air and maintained at subconfluent densities in the growth medium. For cell proliferation experiments, DMEM supplemented with 15% FBS was used. The medium was replaced with DMEM supplemented with 3% FBS for differentiation experiments.

For the experiments using inhibitors of PKB/Akt (LY294002 20 μM) and ERK1/2 (PD98059 10 μM) mediated signaling, cells were treated with the appropriate inhibitor 30 min before Na_2_WO_4_ administration, and the inhibitor was maintained during the incubation period. Glycated bovine albumin (AGEs) was obtained in our laboratory in vitro by incubating bovine serum albumin with glucose−6 phosphate as previously described [44].

Cell lines were provided by the University of Granada (Spain) Cell Culture Facility.

### 3.2. Cell Neurite Outgrowth Assays and Proliferation

Neuro2a or SH-SY5Y cells were incubated in differentiation medium for 48 or 72 h, respectively, in the absence or presence of Na_2_WO_4_ (0−1 mM), and neurite outgrowth was observed under a fluorescent phase-contrast light microscope (Olympus CKX41, Tokyo, Japan) at a magnification of 200×. Images were taken, and the numbers of undifferentiated cells and neurites were counted using ImageJ (National Institutes of Health, Bethesda, MD, USA, https://imagej.net/ij/) [45]. Neurites are defined as processes with lengths equivalent to one or more diameters of the cell body. The percentage of cells bearing neurites was calculated as the percentage of the number of neurites divided by the total number of cells.

For proliferation assays, Neuro2a or SH-SY5Y cells were incubated in complete medium for 48 or 72 h, respectively, in the absence or presence of Na_2_WO_4_ (0−1 mM). Cell proliferation was measured using 3-(4,5-dimethylthiazol−2-yl)−2,5-diphenyl−2H-tetrazolium bromide (MTT), which is reduced by cell dehydrogenases, allowing a correlation between cellular metabolic activity and the number of viable cells in culture.

### 3.3. Cell Cycle Analysis

Cells were incubated under the same conditions to measure neurite outgrowth. At different time points, cells were scraped and pelleted at 600× *g*/10 min. Cells were re-suspended in 200 µL PBS to obtain a suspension of single cells. Next, 2 mL of ice-cold 70% ethanol was added, vigorously mixed, and left for at least 30 min at −20 °C. Cells were collected via centrifugation and resuspended in 800 µL PBS to avoid clumping. One hundred microliters RNase (1 mg/mL) and 100 µL propidium iodide (400 µg/mL) were added, and the suspension was incubated at 37 °C for 30 min. The cell cycle was analyzed using an argon-ion laser tuned to 488 nm with forward and orthogonal light scattering and red fluorescence. Cell cycle analysis was determined in the Flow Cytometry Service (University of Granada, Spain).

### 3.4. Protein Analysis

Cells were incubated in the absence or presence of Na_2_WO_4_ for 30 min or 24 h to determine the expression and phosphorylation status of proteins involved in neuritogenesis. The plates were flash-frozen in liquid nitrogen and processed as previously described [19].

Protein concentration was measured using the bicinchoninic acid method. Proteins (40 μg) were separated using SDS-PAGE, transferred to nitrocellulose membranes, and subjected to immunoblotting with specific antibodies. To assess the degree of phosphorylation of key kinases, antibodies against phosphorylated proteins were used. GAPDH or actin were used as loading controls to determine the expression of other proteins. Immunoblotting was performed using fluorescent secondary antibodies (Bio-Rad, Madrid, Spain).

### 3.5. RNA Extraction and RT-qPCR

Cells were incubated in the absence or presence of Na_2_WO_4_ for 24 h (Neuro2a) or 72 h (SH-SY5Y). RNA was purified using a Gene JET RNA purification kit (Thermo Fisher, Madrid, Spain) following the manufacturer’s instructions.

The amount of total RNA was measured using a NanoVue Plus Spectrophotometer (GE Healthcare Life Sciences, Madrid, Spain), and its quality was verified using 1% agarose gel electrophoresis. One microgram of total RNA from each sample was reverse transcribed using an iScript cDNA Synthesis Kit (Bio-Rad, Madrid, Spain). SsoAdvanced Universal SYBR Green Supermix (Bio-Rad, Madrid, Spain) was used for qPCR, and the results were analyzed using the CFX Maestro software. The oligonucleotides used in this study are listed in Supplemental Materials Table S1.

### 3.6. MEF2D Expression Vectors, Reporter Gene Constructs, and Transcriptional Assays

The rat MEF2D coding sequence was amplified using Pfu polymerase from rat muscle cell cDNA using the oligonucleotides MEF2Df and MEF2Dr (see Supplemental Materials Table S2), including the *Bgl*II and *Sal*I restriction sites. The PCR product was cloned into the pJET 1.2 vector (Thermo Fisher Scientific, Madrid, Spain) and then digested with restriction enzymes to clone it into the pEGFP-C1 eukaryotic expression vector (Clontech Laboratories, Mountain View, CA, USA). The new plasmid was termed pEGFP-MEF2D. This plasmid encodes a GFP-MEF2D fusion protein expressed at the cell’s nucleus. For the eukaryotic expression of MEF2D alone, the same insert was cloned into the pEGFP-N3 plasmid and termed pMEF2D. Because the reverse oligonucleotide used for amplification contains a STOP signal, only the MEF2D protein is translated from this plasmid. 

To analyze MEF2D-dependent transcription in cells lacking MEF2D protein expression (CHO-k1 cells), the pMEF2D plasmid was co-transfected with pMEF2x4 Eb1 Luc (a kindly gift of Prof. Brian Black, UCSF, USA), a plasmid that contains four copies of the MEF2 binding domain in tandem [46]. To generate a transcription reporter system independent of the cell background (Neuro2a and SH-SY5Y cells), pCMV-BD and pFR-Luc plasmids (from the Mammalian Two-Hybrid Assay Kit, Stratagene, La Joya, CA, USA) were used. The pMEF2D insert was digested with *Bgl*II and *Sal*I and cloned into the pCMV-BD plasmid to generate the plasmid pCMV-BD-MEF2D, which expresses the DNA binding domain of GAL4 fused to MEF2D. For this plasmid, cells were co-transfected with a pFR-Luc plasmid containing four copies of the yeast GAL4 binding domain in tandem.

Site-directed mutagenesis of either pEGFP-MEF2D, pMEF2D, or pCMV-BD-MEF2D was carried out as described previously [47] to introduce mutations in the MEF2D coding sequence. The oligonucleotides used for site-directed mutagenesis are also provided in Supplemental Materials Table S2. The sequence of the generated plasmids was confirmed by automatic sequencing using universal primers. 

For the analysis of luciferase activity, cells at 80–90% confluence were transiently transfected using LipofectAMINE2000 as described by the manufacturer. The DNA mixture consisted of the corresponding luciferase reporter and reference plasmid, pRL-TK (ratio 95:5). Cells were incubated in the presence or absence of Na_2_WO_4_ for 24 h. Luciferase activity was determined using the dual-luciferase method (Promega, Madrid, Spain) in a luminometer (Sirius L, Berthold Technologies, Bad Wildbad, Germany), and results were standardized for *Renilla* luciferase. Data were expressed as relative changes in luciferase activity normalized to a value of 100% to facilitate comparisons of expression levels. 

In addition, constructs containing the coding sequence of MEF2, whether mutated or not, and fused to the green fluorescent protein (eGFP) were used to transfect CHO-k1 or Neuro2a cells and study the effect of Na_2_WO_4_ on MEF2D protein stability using a cycloheximide chase assay [48]. In brief, these cells were transfected with the corresponding plasmids. Twenty-four hours after transfection, cells were incubated in growth media in the presence or absence of Na_2_WO_4_ in the presence of 100 µM cycloheximide to block protein synthesis. After adding cycloheximide, fluorescence images (Olympus CKX41) were captured at 0, 1, 3, 6, 12, and 24 h, and the eGFP intensity of the fluorescence of individual cells was analyzed using ImageJ [45]. Fluorescence values were normalized to time 0 under each condition.

### 3.7. Senescence

For the analysis of the expression of β galactosidase (SA-β-gal) as a marker of senescence [49], cells were incubated in the absence or presence of Na_2_WO_4_ for 24 h (Neuro2a) or 72 h (SH-SY5Y). The samples were washed twice with PBS, fixed with 4% formaldehyde for 3 min at room temperature, and washed again twice with PBS. SA-β-gal staining solution (1 mg/mL 5-bromo−4-chloro−3-indolyl-beta-d-galactopyranoside (X-gal), citric acid/sodium phosphate buffer (pH 6.0), 5 mM potassium ferricyanide, 150 mM NaCl, and 2 mM MgCl_2_) was added, and cells were incubated with the staining solution at 37 °C for 12–16 h. After the blue color had fully developed, the cells were washed with PBS. Blue SA-β-gal-positive cells were counted under a microscope, and the percentage of blue cells bearing or not neurites was calculated as a percentage. 

### 3.8. Determination of Protein Synthesis and 2-Deoxy-D-[1−^3^H]glucose Uptake

Cells were treated with Na_2_WO_4_ for 2 h in normal culture media (10% FBS) and 0.8 mmol/l Tyr. They were then spiked with 1 μCi/ml of L-(ring−3,5−^3^H)-Tyr (PerkinElmer, Waltham, MA, USA) and incubated for 1 h. Protein synthesis was measured as described [18]. Data were calculated as dpm/μg of proteins. Neuro2a cells were incubated with Na_2_WO_4_ for 24 h, and measurement of 2-deoxy-D-[1−^3^H]glucose (2-DG) uptake was taken as described [14].

### 3.9. Statistical Analysis

Results are expressed as the mean ± SEM. Statistical analysis was performed using one-way or two-way ANOVA followed by Tukey’s test as appropriate. *p* < 0.05 was considered statistically significant.

## Data Availability

The original contributions presented in the study are included in the article and supplementary material. Further inquiries can be directed to the corresponding author.

## Supplementary Materials

The following supporting information can be downloaded at https://www.mdpi.com/article/10.3390/ijms25179150/s1.

## References

[1] Hrvoj-Mihic B., Bienvenu T., Stefanacci L., Muotri A.R., Semendeferi K. (2013). Evolution, development, and plasticity of the human brain: From molecules to bones. Front. Hum. Neurosci..

[2] Liu H.H., Jan Y.N. (2020). Mechanisms of neurite repair. Curr. Opin. Neurobiol..

[3] Read D.E., Gorman A.M. (2009). Involvement of Akt in neurite outgrowth. Cell Mol. Life Sci..

[4] Chong Z.Z., Shang Y.C., Wang S., Maiese K. (2012). A Critical Kinase Cascade in Neurological Disorders: PI 3-K., Akt, and mTOR. Future Neurol..

[5] El Ouaamari Y., Van den Bos J., Willekens B., Cools N., Wens I. (2023). Neurotrophic Factors as Regenerative Therapy for Neurodegenerative Diseases: Current Status, Challenges and Future Perspectives. Int. J. Mol. Sci..

[6] Park S.J., Jin M.L., An H.K., Kim K.S., Ko M.J., Kim C.M., Choi Y.W., Lee Y.C. (2015). Emodin induces neurite outgrowth through PI3K/Akt/GSK−3beta-mediated signaling pathways in Neuro2a cells. Neurosci. Lett..

[7] Wu P.Y., Lin Y.C., Chang C.L., Lu H.T., Chin C.H., Hsu T.T., Chu D., Sun S.H. (2009). Functional decreases in P2X7 receptors are associated with retinoic acid-induced neuronal differentiation of Neuro−2a neuroblastoma cells. Cell Signal..

[8] Yanaka N., Nogusa Y., Fujioka Y., Yamashita Y., Kato N. (2007). Involvement of membrane protein GDE2 in retinoic acid-induced neurite formation in Neuro2A cells. FEBS Lett..

[9] Shipley M.M., Mangold C.A., Szpara M.L. (2016). Differentiation of the SH-SY5Y Human Neuroblastoma Cell Line. J. Vis. Exp..

[10] Xicoy H., Wieringa B., Martens G.J. (2017). The SH-SY5Y cell line in Parkinson’s disease research: A systematic review. Mol. Neurodegener..

[11] Maiuolo J., Costanzo P., Masullo M., D’Errico A., Nasso R., Bonacci S., Mollace V., Oliverio M., Arcone R. (2023). Hydroxytyrosol-Donepezil Hybrids Play a Protective Role in an In Vitro Induced Alzheimer’s Disease Model and in Neuronal Differentiated Human SH-SY5Y Neuroblastoma Cells. Int. J. Mol. Sci..

[12] Bertinat R., Nualart F., Li X., Yanez A.J., Gomis R. (2015). Preclinical and Clinical Studies for Sodium Tungstate: Application in Humans. J. Clin. Cell. Immunol..

[13] Zafra D., Nocito L., Dominguez J., Guinovart J.J. (2013). Sodium tungstate activates glycogen synthesis through a non-canonical mechanism involving G-proteins. FEBS Lett..

[14] Giron M.D., Sevillano N., Vargas A.M., Dominguez J., Guinovart J.J., Salto R. (2008). The glucose-lowering agent sodium tungstate increases the levels and translocation of GLUT4 in L6 myotubes through a mechanism associated with ERK1/2 and MEF2D. Diabetologia.

[15] Fernandez-Alvarez J., Barbera A., Nadal B., Barcelo-Batllori S., Piquer S., Claret M., Guinovart J.J., Gomis R. (2004). Stable and functional regeneration of pancreatic beta-cell population in nSTZ-rats treated with tungstate. Diabetologia.

[16] Dominguez J.E., Munoz M.C., Zafra D., Sanchez-Perez I., Baque S., Caron M., Mercurio C., Barbera A., Perona R., Gomis R. (2003). The antidiabetic agent sodium tungstate activates glycogen synthesis through an insulin receptor-independent pathway. J. Biol. Chem..

[17] Amigo-Correig M., Barcelo-Batllori S., Piquer S., Soty M., Pujadas G., Gasa R., Bortolozzi A., Carmona M.C., Gomis R. (2011). Sodium tungstate regulates food intake and body weight through activation of the hypothalamic leptin pathway. Diabetes Obes. Metab..

[18] Salto R., Vilchez J.D., Cabrera E., Guinovart J.J., Giron M.D. (2014). Activation of ERK by sodium tungstate induces protein synthesis and prevents protein degradation in rat L6 myotubes. FEBS Lett..

[19] Salto R., Vilchez J.D., Giron M.D., Cabrera E., Campos N., Manzano M., Rueda R., Lopez-Pedrosa J.M. (2015). beta-Hydroxy-beta-Methylbutyrate (HMB) Promotes Neurite Outgrowth in Neuro2a Cells. PLoS ONE.

[20] Wang J.L., Wang J.J., Cai Z.N., Xu C.J. (2018). The effect of curcumin on the differentiation, apoptosis and cell cycle of neural stem cells is mediated through inhibiting autophagy by the modulation of Atg7 and p62. Int. J. Mol. Med..

[21] Galderisi U., Jori F.P., Giordano A. (2003). Cell cycle regulation and neural differentiation. Oncogene.

[22] Hindley C., Philpott A. (2012). Co-ordination of cell cycle and differentiation in the developing nervous system. Biochem. J..

[23] Si Z., Sun L., Wang X. (2021). Evidence and perspectives of cell senescence in neurodegenerative diseases. Biomed. Pharmacother..

[24] Dickey C.A., De Mesquita D.D., Morgan D., Pennypacker K.R. (2004). Induction of memory-associated immediate early genes by nerve growth factor in rat primary cortical neurons and differentiated mouse Neuro2A cells. Neurosci. Lett..

[25] Wang X., Wang Z., Yao Y., Li J., Zhang X., Li C., Cheng Y., Ding G., Liu L., Ding Z. (2011). Essential role of ERK activation in neurite outgrowth induced by alpha-lipoic acid. Biochim. Biophys. Acta.

[26] Wang Z., Wang J., Li J., Wang X., Yao Y., Zhang X., Li C., Cheng Y., Ding G., Liu L. (2011). MEK/ERKs signaling is essential for lithium-induced neurite outgrowth in N2a cells. Int. J. Dev. Neurosci..

[27] Zoungrana L.I., Krause-Hauch M., Wang H., Fatmi M.K., Bates L., Li Z., Kulkarni P., Ren D., Li J. (2022). The Interaction of mTOR and Nrf2 in Neurogenesis and Its Implication in Neurodegenerative Diseases. Cells.

[28] Coelho P., Fao L., Mota S., Rego A.C. (2022). Mitochondrial function and dynamics in neural stem cells and neurogenesis: Implications for neurodegenerative diseases. Ageing Res. Rev..

[29] Peng W., Tan C., Mo L., Jiang J., Zhou W., Du J., Zhou X., Liu X., Chen L. (2021). Glucose transporter 3 in neuronal glucose metabolism: Health and diseases. Metabolism.

[30] Agostini M., Romeo F., Inoue S., Niklison-Chirou M.V., Elia A.J., Dinsdale D., Morone N., Knight R.A., Mak T.W., Melino G. (2016). Metabolic reprogramming during neuronal differentiation. Cell Death Differ..

[31] Akhtar M.W., Kim M.S., Adachi M., Morris M.J., Qi X., Richardson J.A., Bassel-Duby R., Olson E.N., Kavalali E.T., Monteggia L.M. (2012). In vivo analysis of MEF2 transcription factors in synapse regulation and neuronal survival. PLoS ONE.

[32] Shi L., Li B., Chen G., Huang Y., Tian Z., Zhang L., Tian L., Fu Q. (2022). MEF2D Participates in Microglia-Mediated Neuroprotection in Cerebral Ischemia-Reperfusion Rats. Shock.

[33] Wang N., Yang W., Li L., Tian M. (2020). MEF2D upregulation protects neurons from oxygen-glucose deprivation/re-oxygenation-induced injury by enhancing Nrf2 activation. Brain Res..

[34] Chen X., Gao B., Ponnusamy M., Lin Z., Liu J. (2017). MEF2 signaling and human diseases. Oncotarget.

[35] Lam B.Y., Chawla S. (2007). MEF2D expression increases during neuronal differentiation of neural progenitor cells and correlates with neurite length. Neurosci. Lett..

[36] Lisek M., Przybyszewski O., Zylinska L., Guo F., Boczek T. (2023). The Role of MEF2 Transcription Factor Family in Neuronal Survival and Degeneration. Int. J. Mol. Sci..

[37] Gregoire S., Tremblay A.M., Xiao L., Yang Q., Ma K., Nie J., Mao Z., Wu Z., Giguere V., Yang X.J. (2006). Control of MEF2 transcriptional activity by coordinated phosphorylation and sumoylation. J. Biol. Chem..

[38] Yamada M., Nagasaki S.C., Suzuki Y., Hirano Y., Imayoshi I. (2020). Optimization of Light-Inducible Gal4/UAS Gene Expression System in Mammalian Cells. iScience.

[39] Kuhla A., Ludwig S.C., Kuhla B., Munch G., Vollmar B. (2015). Advanced glycation end products are mitogenic signals and trigger cell cycle reentry of neurons in Alzheimer’s disease brain. Neurobiol. Aging.

[40] Schmidt A., Kuhla B., Bigl K., Munch G., Arendt T. (2007). Cell cycle related signaling in Neuro2a cells proceeds via the receptor for advanced glycation end products. J. Neural. Transm..

[41] Ren X., Ma H., Qiu Y., Liu B., Qi H., Li Z., Kong H., Kong L. (2015). The downregulation of thioredoxin accelerated Neuro2a cell apoptosis induced by advanced glycation end product via activating several pathways. Neurochem. Int..

[42] Reddy Addi U., Jakhotia S., Reddy S.S., Reddy G.B. (2022). Age-related neuronal damage by advanced glycation end products through altered proteostasis. Chem. Biol. Interact..

[43] Souza C.G., Riboldi B.P., Hansen F., Moreira J.D., Souza D.G., de Assis A.M., Brum L.M., Perry M.L., Souza D.O. (2013). Chronic sulforaphane oral treatment accentuates blood glucose impairment and may affect GLUT3 expression in the cerebral cortex and hypothalamus of rats fed with a highly palatable diet. Food Funct..

[44] Giron-Gonzalez M.D., Morales-Portillo A., Salinas-Castillo A., Lopez-Jaramillo F.J., Hernandez-Mateo F., Santoyo-Gonzalez F., Salto-Gonzalez R. (2014). Engineered glycated amino dendritic polymers as specific nonviral gene delivery vectors targeting the receptor for advanced glycation end products. Bioconjug. Chem..

[45] Schneider C.A., Rasband W.S., Eliceiri K.W. (2012). NIH Image to ImageJ: 25 years of image analysis. Nat. Methods.

[46] Khiem D., Cyster J.G., Schwarz J.J., Black B.L. (2008). A p38 MAPK-MEF2C pathway regulates B-cell proliferation. Proc. Natl. Acad. Sci. USA.

[47] Kucinska M., Giron M.D., Piotrowska H., Lisiak N., Granig W.H., Lopez-Jaramillo F.J., Salto R., Murias M., Erker T. (2016). Novel Promising Estrogenic Receptor Modulators: Cytotoxic and Estrogenic Activity of Benzanilides and Dithiobenzanilides. PLoS ONE.

[48] Kao S.H., Wang W.L., Chen C.Y., Chang Y.L., Wu Y.Y., Wang Y.T., Wang S.P., Nesvizhskii A.I., Chen Y.J., Hong T.M. (2015). Analysis of Protein Stability by the Cycloheximide Chase Assay. Bio Protoc..

[49] Itahana K., Itahana Y., Dimri G.P. (2013). Colorimetric detection of senescence-associated beta galactosidase. Methods Mol. Biol..

