# Supplementary material for: Sodium Tungstate Promotes Neurite Outgrowth and Confers Neuroprotection in Neuro2a and SH-SY5Y Cells
Source: Int J Mol Sci. 2024 Aug 23;25(17):9150. doi: 10.3390/ijms25179150 (PMC11394838; doi:10.3390/ijms25179150)
Supplement: Supplementary file 1 [file ijms-25-09150-s001.zip › ijms-3127110-supplementary.pdf]

International Journal of
Molecular Sciences

MDPI

Supplementary Materials

Sodium Tungstate Promotes Neurite Outgrowth and Confers Neuroprotection in Neuro2a and SH-SY5Y Cells

Nora Montero-Martin, María D. Girón *, José D. Vílchez and Rafael Salto

Department of Biochemistry and Molecular Biology II, School of Pharmacy, University of Granada, E18071 Granada, Spain; nora84@ugr.es (N.M.-M.); damasovr@gmail.com (J.D.V.); rsalto@ugr.es (R.S.)
* Correspondence: mgiron@ugr.es

Electronic Supplementary Materials

1.- **Table S1.** *Oligonucleotides for qPCR used in this article.* Page 2

2.- **Table S2.** *Oligonucleotides used for the cloning and mutagenesis of rat MEF2D.* Page 2

3.- **Figure S1.** *Representative images of Na_2WO_4 effects on neurite outgrowth in Neuro2a (a) and SH-SY5Y (b) cells.* Page 3

4.- **Figure S2** *Flow cytometry analysis of the cell cycle.* Page 4

5. **Figure S3** *Effects of Na_2WO_4 effects on cell viability measured as LDH release.* Page 5

6. **Figure S4**. *Na_2WO_4-induced neurite outgrowth is mediated by PI3K/Akt and ERK1/2 signaling pathways in Neuro2a cells.* Page 5

7. **Figure S5.** *Na_2WO_4 reverses the effects of advanced glycation end products (AGEs) on neurons.* Page 6

8. **Figure S6.** *Na_2WO_4 normalizes the cell cycle on cells incubated with advanced glycation end products (AGEs) on neurons.* Page 7

9. **Figure S7.** *Na_2WO_4 normalizes the procaspase 3 processing on cells incubated with advanced glycation end products (AGEs) on neurons.* Page 8

Citation: Montero-Martin, N.; Gíron, M.D.; Vílchez, J.D.; Salto, R. Sodium Tungstate Promotes Neurite Outgrowth and Confers Neuroprotection in Neuro2a and SH-SY5Y Cells. *Int. J. Mol. Sci.* **2024**, *25*, 9150. https://doi.org/10.3390/ijms25179150

Academic Editors: Anna-Leena Sirén and Aleksey Zaitsev

Received: 11 July 2024
Revised: 13 August 2024
Accepted: 21 August 2024
Published: 26 August 2024

Copyright: © 2024 by the authors. Submitted for possible open access publication under the terms and conditions of the Creative Commons Attribution (CC BY) license (https://creativecommons.org/licenses/by/4.0/).

Table S1. *qPCR Oligonucleotides used for the quantitation of mRNA in this article*. The sequences of the forward and reverse oligonucleotides are shown.

Gene	Specie	Name	Sequence
Actin	Mouse	FM1_Actb	5′-GATGTATGAAGGCTTTGGTC-3′
		RM1_Actb	5′-TGTGCACTTTTATTGGTCTC-3′
	Human	FH1_Actb	5′-GACGACATGGAGAAAATCTG-3′
		RH1_Actb	5′-ATGATCTGGGTCATCTTCTC 3′
Choline O-acetyl transferase	Mouse	FM1_Chat	5′-TCCTCTTAAAAGACTCCACC-3′
		RM1_Chat	5′-GACTTGTCATACCAACGATTC-3′
	Human	FH1_Chat	5′-TCAGTTCTTTGTCTTGGATG-3`
		BH1_Chat	5′-TGGAAGCCATTTTGACTATC-3′
Nurr1	Mouse	FM1_Nurr1	5′-CTATGGTCACAGAGAGACAC-3′
		RM1_Nurr1	5′-GCTTTGAGAACATGGACTAC-3′
	Human	FH1_Nr4a2	5′-GACTATCAAATGAGTGGAGATG-3
		BH1_Nr4a2	5′-GACCTGTATGCTAATCGAAG-3′
Tyrosine Hydroxylase	Mouse	FM1_Th	5′-ATGGAAATGCTGTTCTCAAC-3′
		RM1_Th	5′-GTCTCTAAGTGGTGGATTTTG-3′
	Human	FH1_Th	5′-CAAAATCCACCATCTAGAGAC-3′
		BH1_Th	5′-CTGACACTTTTCTTGGGAAC-3′
MEF2D	Mouse	FM1_Mef2d	5′-ATGAACTGATCACTAGTCCC-3′
		RM1_Mef2d	5′-CCTTCTTCATCAGTCCAAAC-3′

Table S2. *Oligonucleotides used for the cloning and mutagenesis of rat MEF2D*. The sequences of the forward and reverse oligonucleotides are shown. Underlined nucleotides correspond to introduced mutations or new restriction sites.

Name	Sequence	Site
MEF2Df	5′-AGATCTATGGGGAGGAAAAAGATTCAG-3′	*Bgl*II
MEF2Dr	5′-GTCGACTCACTTTAATGTCCAAGTATCC-3′	*Sal*I
MEF2D K439Rf	5′CCCCCACATAAGCATCAGATCTGAACCAGTGTCCCCAAGTCG-3′	*Bgl*II
MEF2D K439Rr	5′-CGACTTGGGGACACTGGTTCAGATCTGATGCTTATGTGGGGG-3′	*Bgl*II
Mef2D S444Af	5′-GCATCAAGTCAGAACCAGTGGCCCCGAGTCGTGAACGCAGCCCTGCACC-3′	*Ava*I
MEF2D S44Ar	5′- GGTGCAGGGCTGCGTTCACGACTCGGGGCCACTGGTTCTGACTTGATGC-3′	*Ava*I

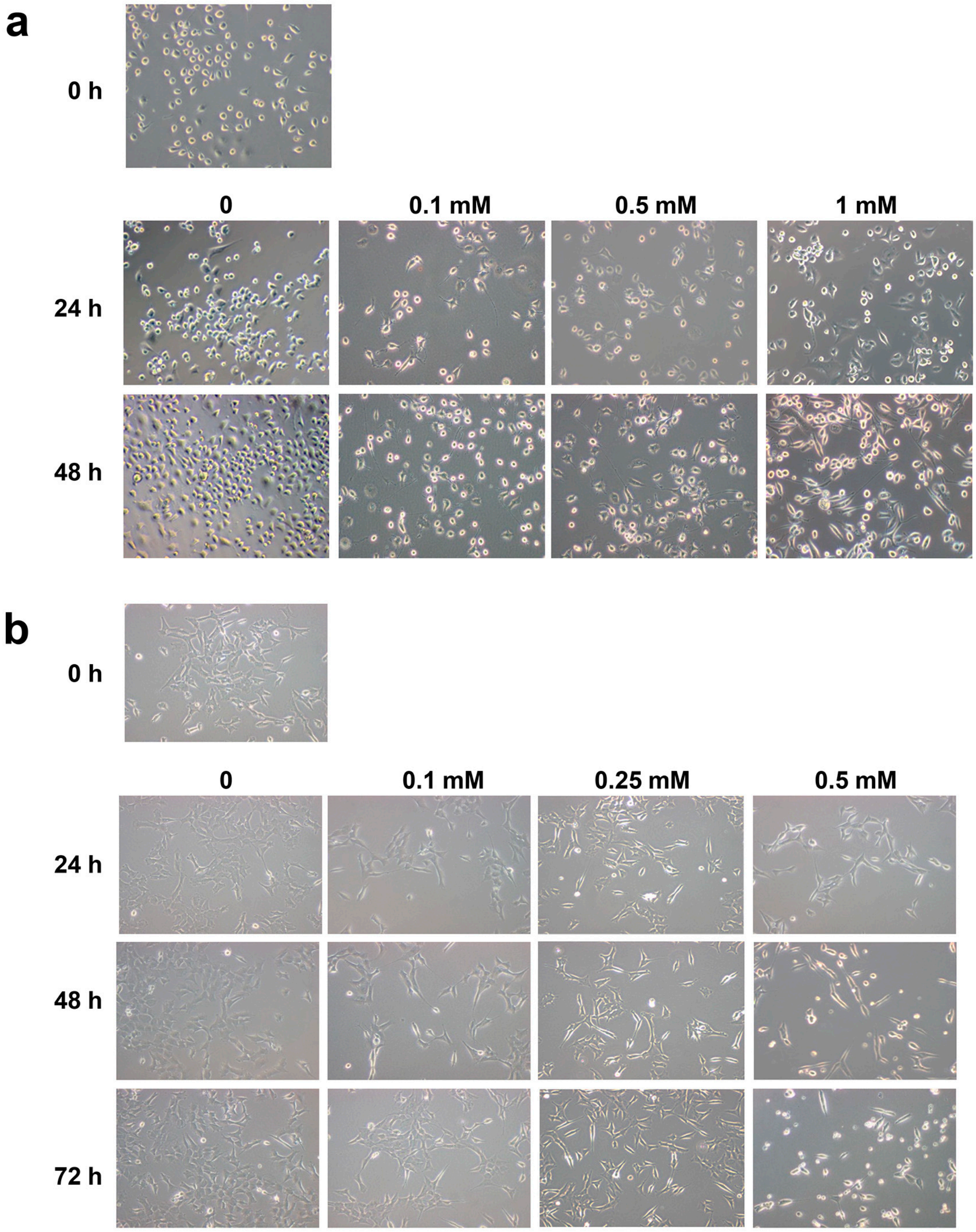

Figure S1. *Representative images of Na_2WO_4 effects on neurite outgrowth in Neuro2a (**a**) and SH-SY5Y (**b**) cells.* Neuro2a **(a)** and SH-SY5Y **(b)** cells were incubated with different concentrations of Na_2WO_4 and neurite outgrowth was determined by visible microscopy.

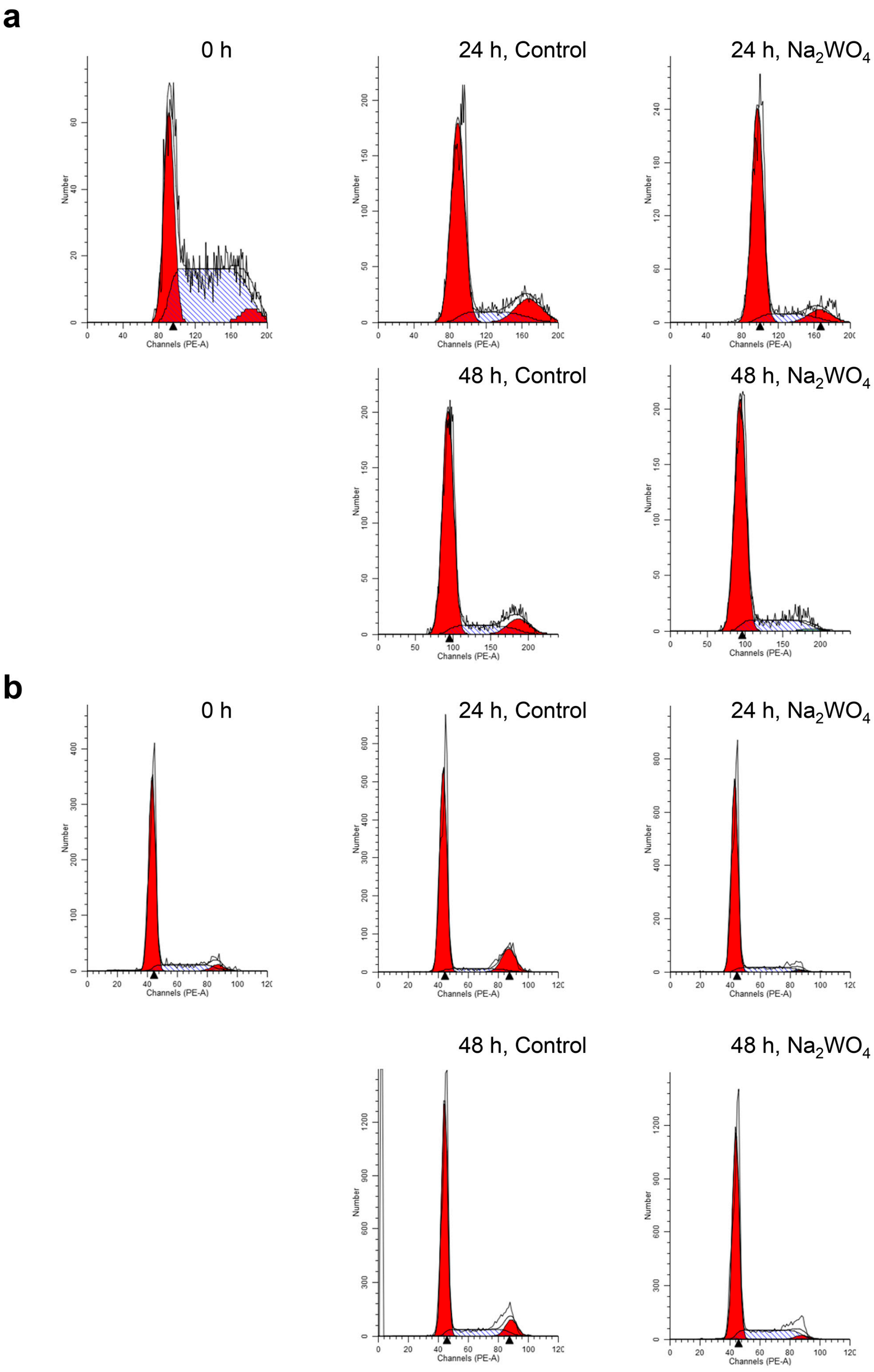

Figure S2. *Flow cytometry analysis of the cell cycle.* Fluorescence histograms of Neuro2a **(a)** and SH-SY5Y **(b)** cells incubated in the absence or presence of Na_2WO_4 (1 mM for Neuro2a and 0.25 mM for SH-SY5Y) at 48 h.

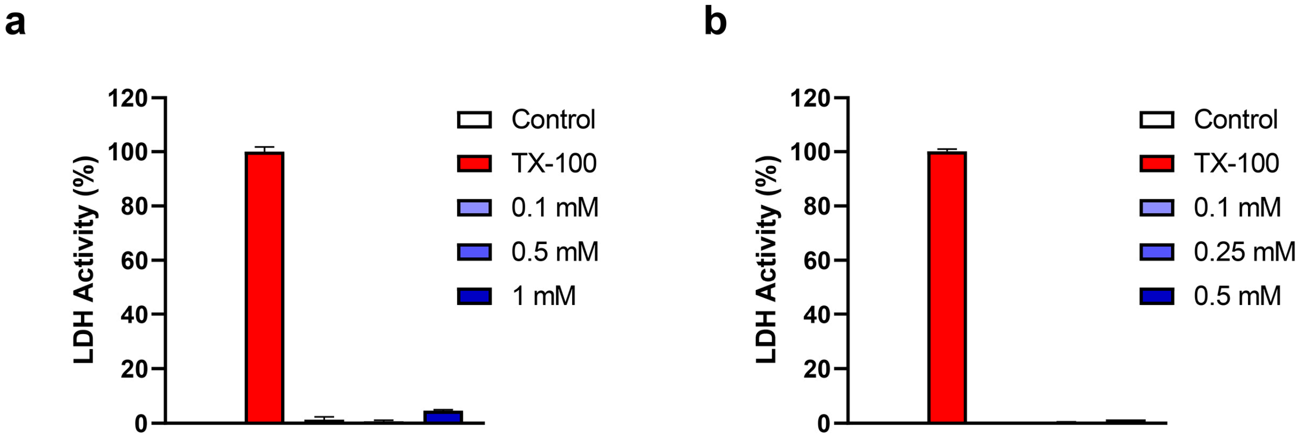

Figure S3. *Effects of Na_2WO_4 effects on cell viability measured as the LDH release.* Neuro2a **(a)** and SH-SY5Y **(b)** cells were incubated in the absence or presence of Na_2WO_4 (48 h for Neuro2a and 0.25 mM for SH-SY5Y) at 48 h.

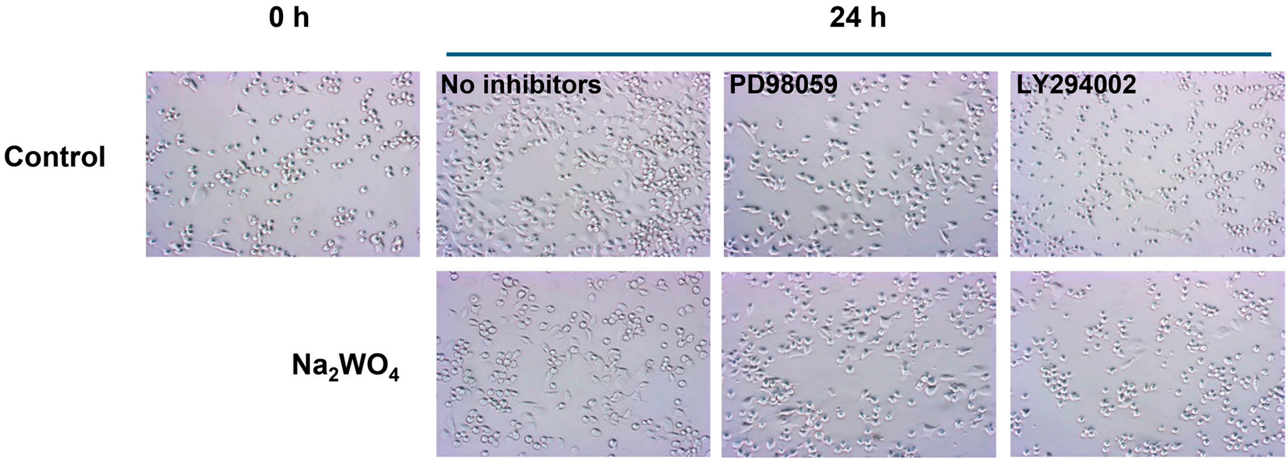

Figure S4. *Na_2WO_4-induced neurite outgrowth is mediated by PI3K/Akt and ERK1/2 signaling pathways in Neuro2a cells.* Neuro2a cells were pre-treated with PD98059 10μM or LY294002 20μM and then treated with 1 mM Na2WO4 for 24 h **(a)** or 30 min **(b)**. Inhibitors were maintained during the experiment. The percentage of differentiated cells was determined by analysis of the cell morphology.

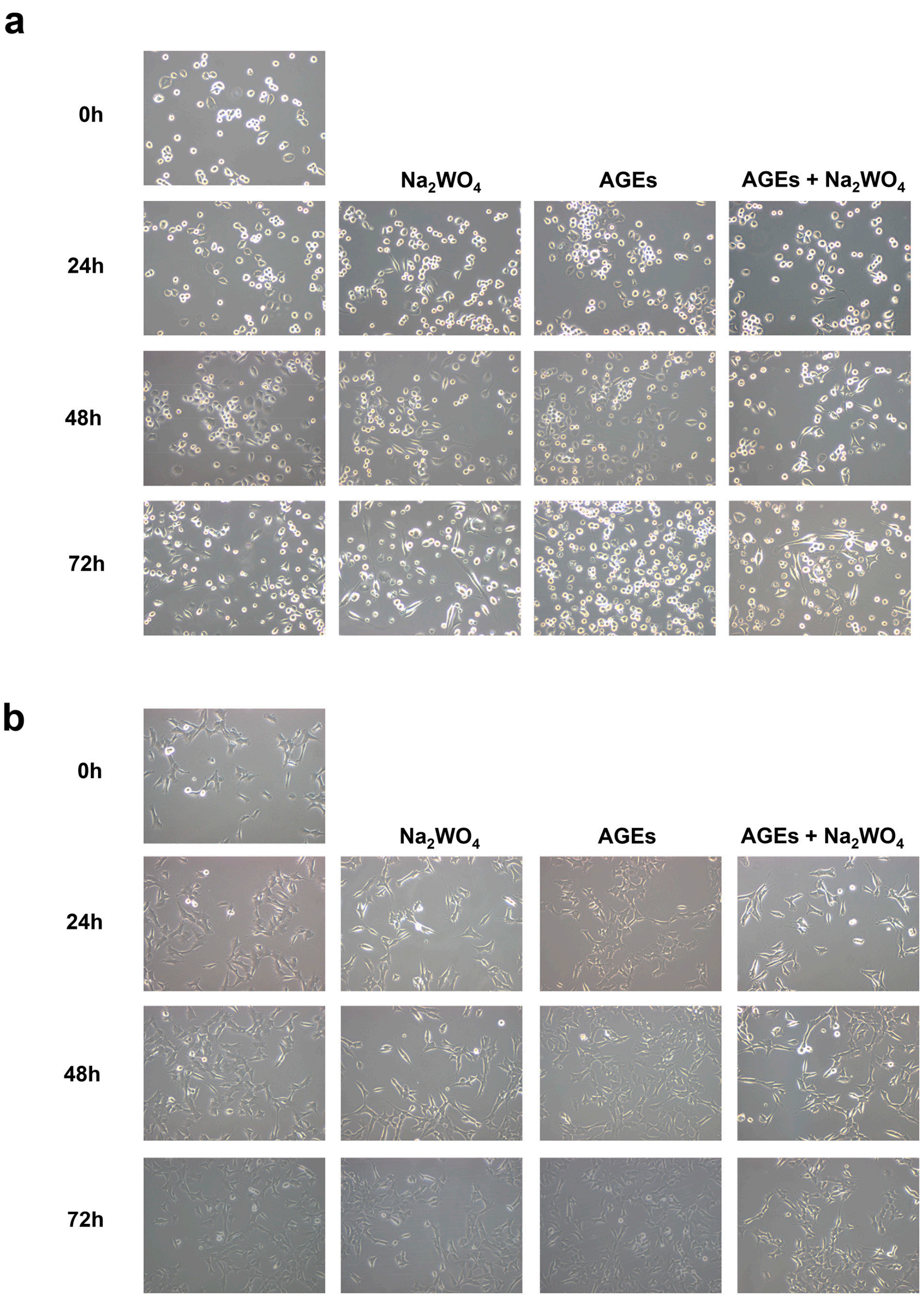

Figure S5. *Na_2WO_4 reverses the effects of advanced glycation end products (AGEs) on neurons.* Neuro 2a and SH-SY5Y cells were incubated with 100 µg/mL AGEs in the absence or presence of Na_2WO_4. **(a)** Cells with neurite outgrowth in Neuro 2a **(a)** or SH-SY5Y cells **(b)** incubated with the effectors for 72 hours. The percentage was determined by analysis of the cell morphology. **(b)** Fluorescence histograms of Neuro2a and SH-SY5Y cells incubated with AGEs in the absence or presence of Na_2WO_4 (1 mM for Neuro2a and 0.25 mM for SH-SY5Y).

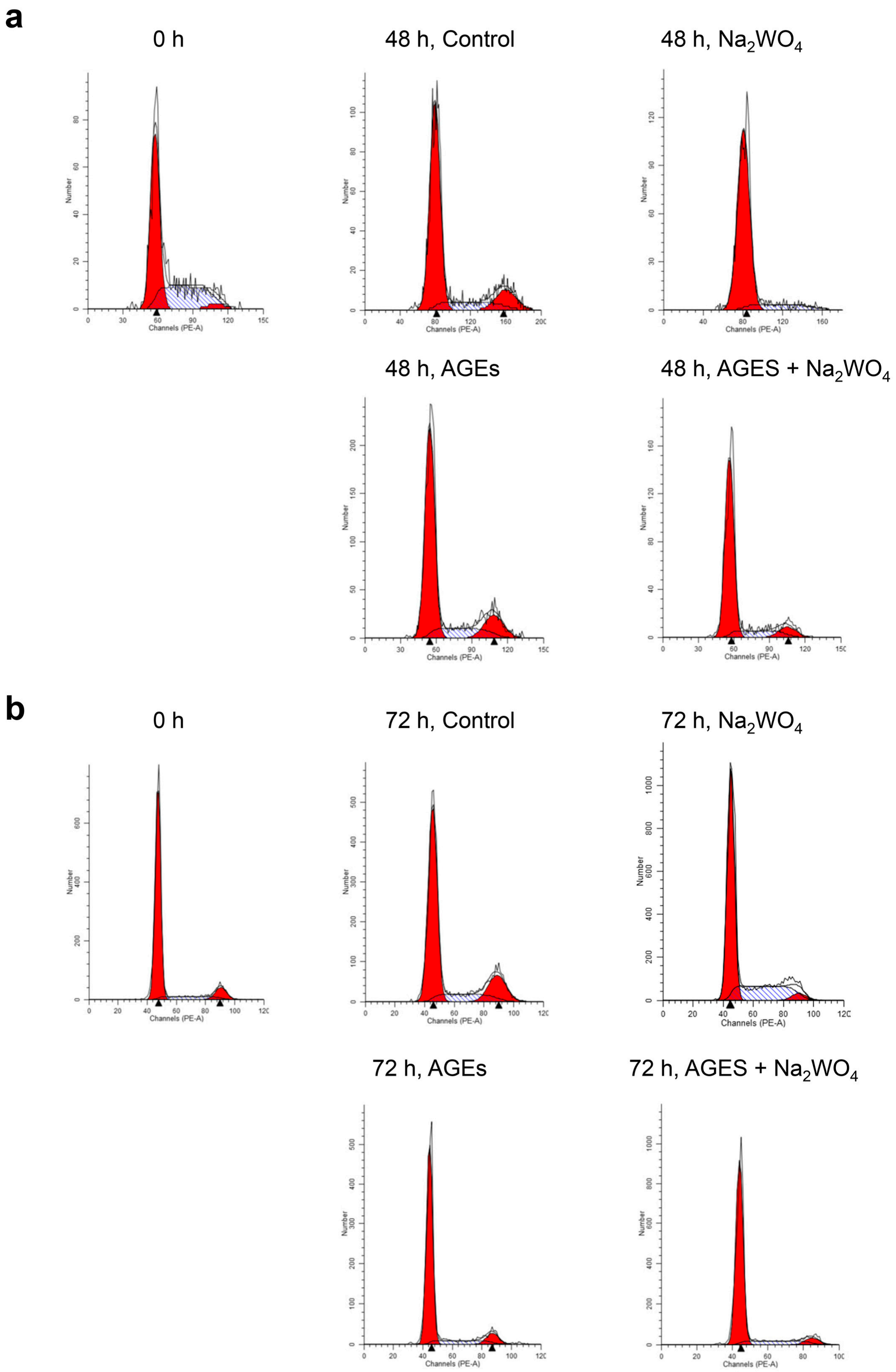

Figure S6. *Na2WO4 normalizes the cell cycle on cells incubated with advanced glycation end products (AGEs) on neurons.* Neuro2a and SH-SY5Y cells were incubated with 100 µg/mL AGEs in the absence or presence of Na_2WO_4. Fluorescence histograms of Neuro2a **(a)** and SH-SY5Y cells **(b)** incubated with AGEs in the absence or presence of Na_2WO_4 (1 mM for Neuro2a and 0.25 mM for SH-SY5Y).

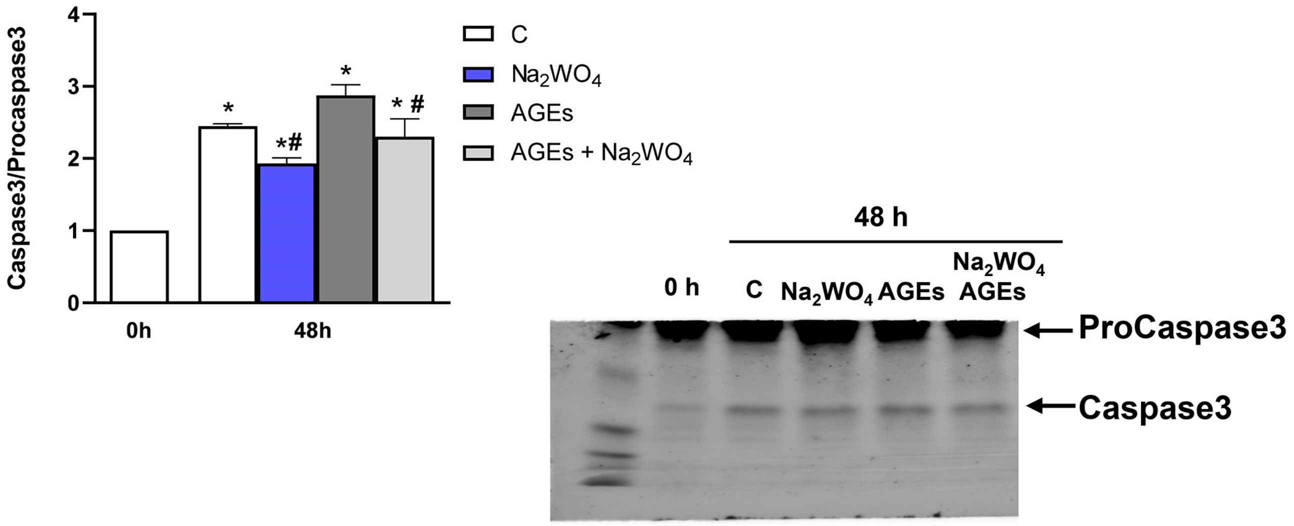

Figure S7. *Na_2WO_4 normalizes the procaspase 3 processing on cells incubated with advanced glycation end products (AGEs) on neurons.* Neuro2a cells were incubated with 100 µg/mL AGEs in the absence or presence of Na_2WO_4 (1 mM). Procaspase and caspase 3 were analyzed by Western blot using antibodies that recognize both forms (Cell Signalling). Results represent means ± SEM (n = 4). *$p<0.05$ vs untreated cells; #$p<0.05$ vs AGEs treated cells.
